# Supplementary material for: Computer-Aided Design for Identifying Anticancer Targets in Genome-Scale Metabolic Models of Colon Cancer
Source: Biology (Basel). 2021 Oct 29;10(11):1115. doi: 10.3390/biology10111115 (PMC8614794; doi:10.3390/biology10111115)
Supplement: Supplementary file 1 [file biology-10-01115-s001.zip › SupplementaryFile1.pdf]

Supplementary Materials for Computer-Aided Design for Identifying Anticancer Targets in Genome-Scale Metabolic Models of Colon Cancer

Chao-Ting Cheng [1], Tsun-YuWang 1, Pei-Rong Chen [1], Wu-Hsiung Wu [1], Jin-Mei Lai [2], Peter Mu-Hsin Chang [3,4], Yi-Ren Hong [5], Chi-Ying F. Huang [6,7] and Feng-Sheng Wang [1,*]

[1] Department of Chemical Engineering, National Chung Cheng University, Chiayi, Taiwan; easonchao1008@gmail.com (C.-T.C.); yu19941120@gmail.com (T.-Y.W.); a6901378tw@gmail.com (P.-R.C.); wwh@cs.ccu.edu.tw (W.-H.W.); chmfsw@ccu.edu.tw (F.-S.W.)

[2] Department of Life Science, Fu-Jen Catholic University, New Taipei City, Taiwan; 062989@mail.fju.edu.tw

[3] Department of Oncology, Taipei Veterans General Hospital, Taipei, Taiwan; ptchang@vghtpe.gov.tw

[4] Faculty of Medicine, National Yang Ming Chiao Tung University, Taipei, Taiwan; ptchang@vghtpe.gov.tw

[5] Department of Biochemistry and Graduate Institute of Medicine, Kaohsiung Medical University, Kaohsiung City, Taiwan; m835016@cc.kmu.edu.tw

[6] Institute of Biopharmaceutical Sciences, National Yang Ming Chiao Tung University, Taipei, Taiwan; cyhuang5@ym.edu.tw

[7] Department of Biotechnology and Laboratory Science in Medicine, National Yang Ming Chiao Tung University, Taipei, Taiwan; cyhuang5@ym.edu.tw

[*] Correspondence: chmfsw@ccu.edu.tw; Tel.: +886 5 2720411x33404

Table S1: Average AE grades for drugs acting on DHODH. Please refer to the DHODH worksheet in the SupplementaryFile2.xlsx file.
Table S2: Average AE grades for drugs acting on HMGCR. Please refer to the HMGCR worksheet in the SupplementaryFile2.xlsx file.
Table S3: The $\log_2$ fold changes of metabolite-flows for the template, identified anticancer target genes, and identified antimetabolites. Please refer to the MetaboliteFlowChange worksheet in the SupplementaryFile3.xlsx file.

Figure S1: The mathematical formulation of the outer optimization problem in IACT framework

Outer optimization problem:

$$
\begin{cases}
\text{The first goal is to measure lethality of cancer cells} \\
\text{for treatment:} \\
\begin{cases}
\text{Fuzzy minimizing the growth rate of cancer cells for treatment} \\
\min\limits_{\delta^{TR},\mathbf{z}} v_{biomass}^{TR} \approx 0
\end{cases} \\
\text{The second goal is to evaluate cell viability of normal cells} \\
\text{due to perturbation:} \\
\begin{cases}
\text{Fuzzy minimizing the pertubed cell as close to zero as possible:} \\
\overline{\min\limits_{\delta^{TR},\mathbf{z}} v_{biomass}^{PB}} \approx 0 \\
\text{Fuzzy maximizing ATP production rate of pertubed cell:} \\
\overline{\max\limits_{\delta^{TR},\mathbf{z}} v_{ATP}^{PB}} \approx v_{ATP}^{\max}
\end{cases} \\
\text{The third goal is to measure flux-pattern variation of perturbed} \\
\text{cell to the CA/BL template:} \\
\begin{cases}
\text{Minimization of similarity ratio of change trend for fluxes and} \\
\text{metabolite-flows to the template:} \\
\min\limits_{\delta^{TR},\mathbf{z}_i} S_F, \min\limits_{\delta^{TR},\mathbf{z}_i} S_M \\
\text{Fuzzy dissimilarity of logarithmic fold change of fluxes and} \\
\text{metabolite-flows to the template:} \\
\overline{\text{dissimilarity}}_{\delta^{TR},\mathbf{z}} L_F \not\approx L_F^{CABL}, \overline{\text{dissimilarity}}_{\delta^{TR},\mathbf{z}} L_M \not\approx L_M^{CABL}
\end{cases} \\
\text{The fourth goal is to meassure flux-pattern of perturbed cell to} \\
\text{the normal:} \\
\begin{cases}
\text{Fzzy similarity of flux and metabolite-flow alterations to the nomal cell:} \\
\overline{\text{similarity}}_{\delta^{TR},\mathbf{z}} v_j \approx v_j^{BL}, \overline{\text{similarity}}_{\delta^{TR},\mathbf{z}} r_m \approx r_m^{BL}
\end{cases}
\end{cases}
\quad \text{(S1-1)}
$$

The fuzzy minimization, $\overline{\min}$, means that the growth rate of cancerous and mutant cells achieves to zero as possible. By contrast fuzzy maximization, $\overline{\max}$, indicates that ATP production rate of the perturbed cells would be as large the upper bound as possible. The fuzzy dissimilarity, $\overline{dissimilarity}$, is a generalized objective function that is different from traditional optimization problems. It is applied for evaluating how a disparity between the fold changes of metabolite-flow or flux-sum for the perturbed cells and the cancer template. A significant disparity (low membership grade) means the flux changes of the perturbed cells are considerable unlike to the

template. This situation implies that the determined target has low metabolic deviation to the normal cells leading to tumorigenesis due to treatment of cancer cells. Fuzzy objectives can be attributed to membership functions in order to convert them into decision criteria so that a fuzzy optimization problem becomes a maximizing decision problem.

Fuzzy objectives in Eq.(S1-1) can be attributed to membership functions in order to convert them into decision criteria so that a fuzzy optimization problem becomes a maximizing decision problem (Hsu and Wang, 2013; Zimmermann, 2010; Massad, et. al., 2008). The transformation of each objective in the outer optimization problem (S1-1) describes as follows:

- The first fuzzy objective is applied to measure lethality of cancer cells for treatment and the second fuzzy objective is to measure cell growth of normal cells due to perturbation for treatment. It indicates that we would like to determine anticancer targets that the influence of cell growth for the perturbed cells is as small as possible.

$$\begin{cases} \text{Fuzzy minimizing the cell growth rate of cancer cells to zero as possible:} \\ \min\limits_{\delta^{TR},\mathbf{z}} v_{biomass}^{TR} \approx 0 \end{cases} \tag{S1-2}$$

$$\begin{cases} \text{Fuzzy minimizing the cell growth of the perturbed cell to zero as possible:} \\ \min\limits_{\delta^{TR},\mathbf{z}} v_{biomass}^{PB} \approx 0 \end{cases} \tag{S1-3}$$

The fuzzy minimizing objectives can elicit a one-side membership function to convert into a decision criterion. A linear membership function is a simplest formulation (Figure S2-1), and expressed as follows:

$$\eta_{bimass}^{TR/PB} = \begin{cases} 1, & \text{if } v_{biomass}^{TR/PB} < v_{biomass}^{LB} \\ \dfrac{v_{biomass}^{UB} - v_{biomass}^{TR/PB}}{v_{biomass}^{UB} - v_{biomass}^{LB}}, & \text{if } v_{biomass}^{LB} \le v_{biomass}^{TR/PB} \le v_{biomass}^{UB} \\ 0, & \text{if } v_{biomass}^{TR/PB} > v_{biomass}^{UB} \end{cases} \tag{S1-3}$$

where the lower and upper bounds, $v_{biomass}^{LB}$ and $v_{biomass}^{UB}$, are provided by the user.

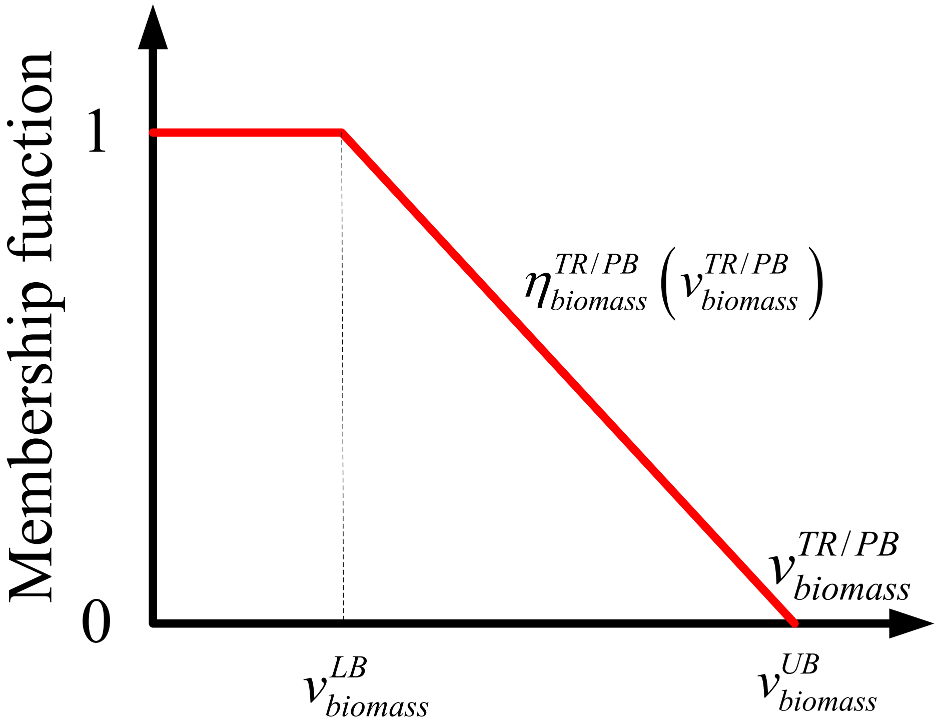

Figure S1-1. A one-side linear membership function for evaluating a fuzzy minimizing objective.

- The third objective is applied to measure cell viability for perturbed cells. It indicates that we would like to find anticancer targets that the ATP production rate of the perturbed can be as close to the normal level as possible.

$$\begin{cases} \text{Fuzzy maximizing ATP production rate of pertubed cell:} \\ \max_{\delta^{TR},\mathbf{z}} v_{ATP}^{PB} \approx v_{ATP}^{BL,\max} \end{cases} \tag{S1-4}$$

The fuzzy maximizing objective is also applied a one-side membership function (Figure S1-2) to convert into a decision criterion as follows:

$$\eta_{ATP}^{PB} = \begin{cases} 0, & \text{if } v_{ATP}^{PB} < v_{ATP}^{LB} \\ \dfrac{v_{ATP}^{PB} - v_{ATP}^{LB}}{v_{ATP}^{UB} - v_{ATP}^{LB}}, & \text{if } v_{ATP}^{LB} \le v_{ATP}^{PB} \le v_{ATP}^{UB} \\ 1, & \text{if } v_{ATP}^{PB} > v_{ATP}^{UB} \end{cases} \tag{S1-5}$$

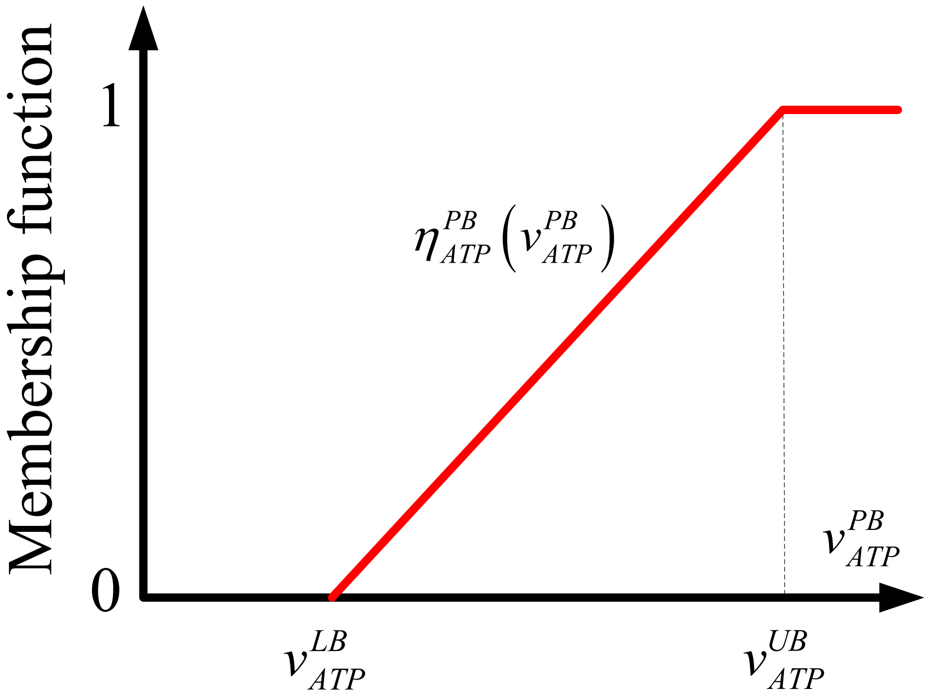

Figure S1-2. A one-side linear membership function for evaluating a fuzzy maximizing objective. The lower and upper bounds, v_{ATP}^{LB} and v_{ATP}^{UB} are provided by a user.

The first goal in the IACT problem is to consider the cell mortality of the treated cells, i.e. η_{CV}^{TR}. The second goal in the IACT problem involves cell growth and ATP production for the perturbed cells. A mean or minimizing value of two membership grades are generally used to measure cell viability. In this study, both evaluations (referred to as mean-min) are combined to compute the second goal by

$$\eta_{CV}^{PB} = \left(\left(\eta_{biomass}^{PB} + \eta_{ATP}^{PB}\right)/2 + \min\left\{\eta_{biomass}^{PB}, \eta_{ATP}^{PB}\right\}\right)/2 \tag{S1-6}$$

A numerical example shown in Table S1-1 is to illustrates a merit of the mean-min evaluation that it can discriminate cases having the identical mean.

Table S1-1. A numerical example to illustrate a mean, min and mean-min evaluation. Mean_min can discriminate the cases having the identical means.

**Case**	$\eta_{biomass}^{PB}$	η_{ATP}^{PB}	$\left(\eta_{biomass}^{PB}+\eta_{ATP}^{PB}\right)/2$	$\min\left\{\eta_{biomass}^{PB},\eta_{ATP}^{PB}\right\}$	η_{CV}^{PB}
**1**	1	0	0.5	0	0.25
**2**	0.5	0.5	0.5	0.5	0.5
**3**	0.4	0.6	0.5	0.4	0.45

- The fourth objectives are to minimize the similarity ratios of fluxes and metabolite-flows to the templates built from cancer and normal models.

$$\begin{cases} \text{Minimization of similarity ratio of change trend for fluxes and} \\ \text{metabolite-flows to the template:} \\ \min\limits_{\delta^{TR},\mathbf{z}_i} S_F, \min\limits_{\delta^{TR},\mathbf{z}_i} S_M \end{cases} \tag{S1-7}$$

The similarity ratios of change trends for fluxes and metabolite-flows are applied to account how many trends of perturbed fluxes/metabolite-flows compared with the normal ones are consistent with the change trends of cancer cells. A perturbed cell gets higher similarity ratios that imply it is more similar distribution pattern to the cancer counterpart. As a result, the perturbed cells would like to lead to tumorigenesis. In contrast, minimizing similarity ratios can prevent tumorigenesis in the IACT problem. For computational purpose, such similarity ratios are replaced by maximizing decision criterion as

$$\eta_{S_F/S_M} = 1 - S_{F/M} \tag{S1-8}$$

The detailed computation of similarity ratio discussed in Wang, et.al. (2020), briefly expresses as follows:

At first, the log2 fold changes of each flux ($L_{f/b}$) and each metabolite-flow ($L_{M,m}$) at perturbed (denoted as PB) and normal (BL) states, and their templates, $L_{f/b}^{T}$ and $L_{M,m}^{T}$, at cancer and normal states are respectively computed as follows:

$$L_{f/b}^{a} = \log_2\left(\frac{v_{f/b}^{PB}}{v_{f/b}^{BL}}\right), a \in \Omega^{Act} \tag{S1-9}$$

$$L_{M,m}^{a} = \log_2\left(\frac{r_m^{PB}}{r_m^{BL}}\right), a \in \Omega^{Act} \quad \text{(S1-10)}$$

$$L_{f/b}^{T} = \log_2\left(\frac{v_{f/b}^{CA}}{v_{f/b}^{BL}}\right) \quad \text{(S1-11)}$$

$$L_{M,m}^{T} = \log_2\left(\frac{r_m^{CA}}{r_m^{BL}}\right) \quad \text{(S1-12)}$$

where Ω^a is the set of discovered anticancer targets, the metabolite-flow or overall synthesis rates (r_m) of the mth metabolite in perturbed, cancer and basal states are respectively computed as follows:

$$r_m^{PB/CA/BL} = \sum_{i \in \Omega^c}\left(\sum_{N_{ij}>0,j} N_{ij} v_{f,j}^{PB/CA/BL} - \sum_{N_{ij}<0,j} N_{ij} v_{b,j}^{PB/CA/BL}\right), m \in \Omega^m \quad \text{(S1-13)}$$

Here Ω^c is the set of metabolites involved in different compartments of the cells, and Ω^m is the set of metabolites. The expression enclosed in brackets indicates the synthesis rate of the ith metabolite that summed the influxes of the forward reactions and backward reactions. The similarity ratios (S_F and S_M) of fluxes and metabolite-flows for the side effect are calculated as follows:

$$S_F = \left(\frac{\sum_{i=1}^{N_f}\left|\mu_i^f\right|}{N_f} + \frac{\sum_{i=1}^{N_b}\left|\mu_i^b\right|}{N_b}\right) \bigg/ 2 \quad \text{(S1-14)}$$

$$S_M = \frac{\sum_{m=1}^{N_m}\left|\mu_m^M\right|}{N_m} \quad \text{(S1-15)}$$

where the similarity indicators ($\mu_i^{f/b/m}$) for each forward and backward reaction, and m^{th} metabolite are defined as

$$\mu_i^{f/b} = \begin{cases} 1, \text{ if } L_{f/b,i} \geq tol_+ \text{ and } L_{f/b.i}^T \geq tol_+ \\ -1, \text{ if } L_{f/b,i} \leq tol_- \text{ and } L_{f/b,i}^T \leq tol_- \\ 0, \text{ otherwise.} \end{cases} \qquad \text{(S1-16)}$$

$$\mu_m^{M} = \begin{cases} 1, \text{ if } L_{M,m} \geq tol_+ \text{ and } L_{M,m}^T \geq tol_+ \\ -1, \text{ if } L_{M,m} \leq tol_- \text{ and } L_{M,m}^T \leq tol_- \\ 0, \text{ otherwise.} \end{cases} \qquad \text{(S1-17)}$$

The tolerances for increase or decrease are defined as $tol_+ = \log_2(1 + \varepsilon)$ and $tol_- = \log_2(1 - \varepsilon)$, respectively, and ε is the percentage of flux alteration. The similarity indicator represents whether the trend of flux fold change for the template and the perturbed cell is consistent or not. Totally, the similarity ratio is the mean for all trends of both models.

- The fifth objectives are applied to measure dissimilarity of fluxes and metabolite-flows for perturbed cell compared to the cancer template, and similarity of fluxes and metabolite-flows for perturbed cell compared to the normal template, respectively.

$$\begin{cases} \text{Fuzzy dissimilarity of logarithmic fold change of fluxes and} \\ \text{metabolite-flows to the cancer template:} \\ \underset{\delta^{TR},\mathbf{z}}{\text{dissimilarity}} L_F \not\approx L_F^{CABL}, \underset{\delta^{TR},\mathbf{z}}{\text{dissimilarity}} L_M \not\approx L_M^{CABL} \\ \text{Fzzy similarity of flux and metabolite-flow alterations to the nomal cell:} \\ \underset{\delta^{TR},\mathbf{z}}{\overline{\text{similarity}}}\, v_j \approx v_j^{BL}, \underset{\delta^{TR},\mathbf{z}}{\overline{\text{similarity}}}\, r_m \approx r_m^{BL} \end{cases} \qquad \text{(S1-18)}$$

Two-side membership functions are used to represent the fuzzy dissimilarity (Figure S1-3) and fuzzy similarity objectives (Figure S1-4), respectively.

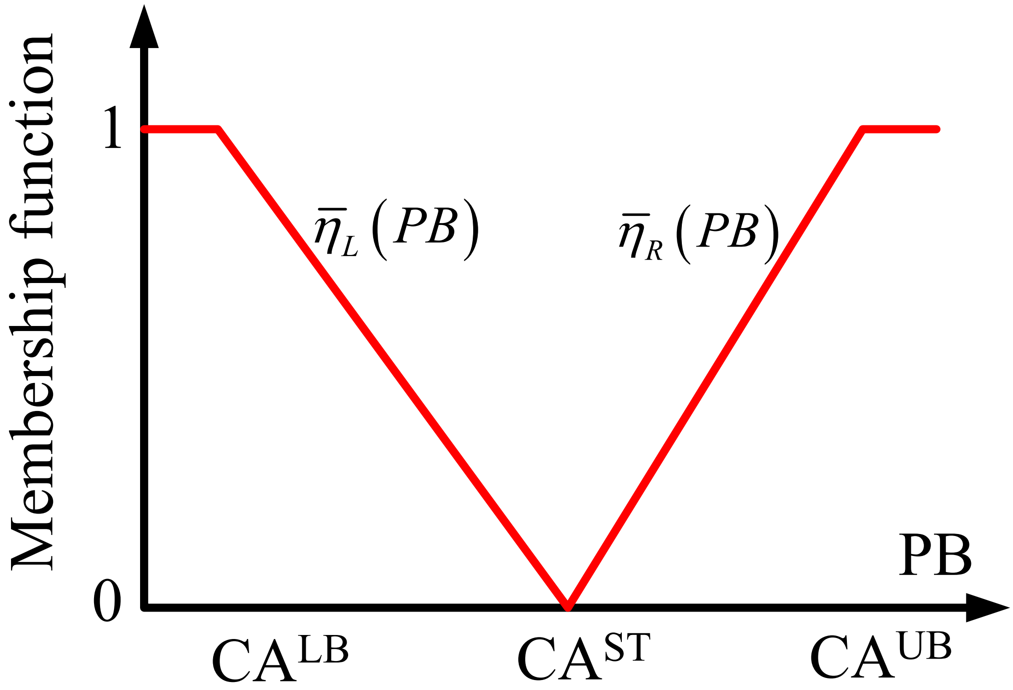

Figure S1-2. A two-side linear membership function for evaluating a fuzzy dissimilarity objective. CA^{LB}, CA^{ST}, and CA^{UB} are, respectively, the lower bound, standard value, and upper bound for fluxes or metabolite flows of cancer templates.

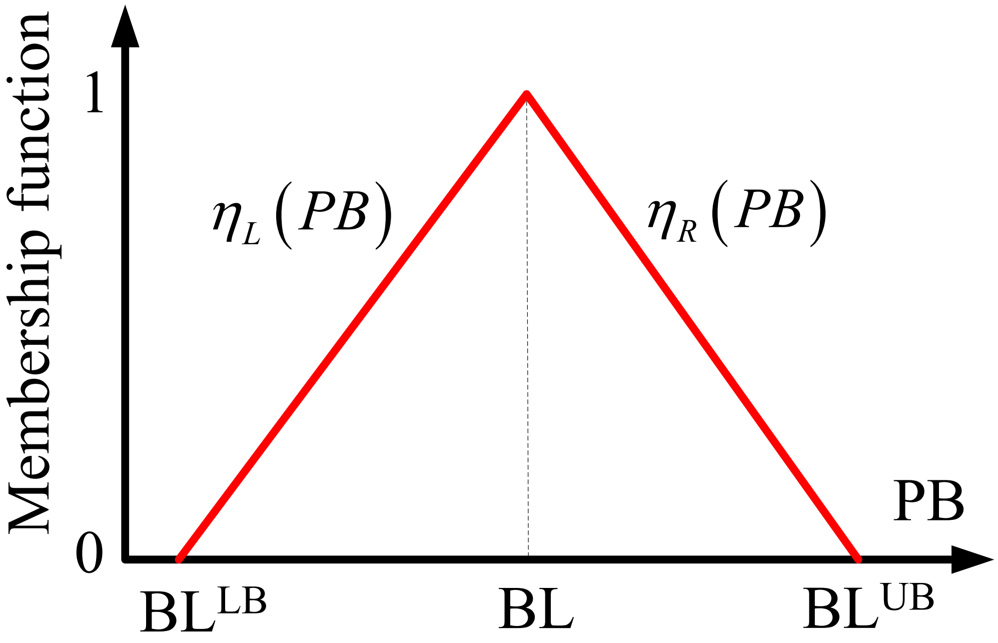

Figure S1-2. A two-side linear membership function for evaluating a fuzzy similarity objective. BL^{LB}, BL^{ST}, and BL^{UB} are, respectively, the lower bound, standard value, and upper bound for fluxes or metabolite flows of normal templates.

Based on fuzzy set theory, fuzzy dissimilarity is a complement of fuzzy similarity. Here, fuzzy similarity for each perturbation is expressed as follows:

Left-hand side membership function:

$$\eta^{L}\left(PB\right)=\frac{PB-BL^{LB}}{BL^{UB}-BL^{LB}}$$

Right-hand side membership function: (S1-19)

$$\eta^{R}\left(PB\right)=\frac{BL^{UB}-PB}{BL^{UB}-BL^{LB}}$$

where the bounds can be provided from clinical experimental data if available. However, up-to-now, genome-scale clinical fluxes are not available. The flux templates computed from the cancer and normal models are provided to estimate the bounds by

Lower bounds of each membership function:

$$LB=\begin{cases}ST/4, & \text{if } ST\geq 0\\ 4ST, & \text{if } ST<0\end{cases}$$

Upper bounds of each membership function: (S1-20)

$$UB=\begin{cases}4ST, & \text{if } ST\geq 0\\ ST/4, & \text{if } ST<0\end{cases}$$

Thus, the membership grade for each metabolic deviation of the perturbed cell is respectively evaluated by

$$\eta_{v/M}=\max\left\{\min\left\{\eta^{L},\eta^{R},1\right\},0\right\} \quad \text{(S1-21)}$$

Based on the complement fuzzy similarity, the membership grade for fuzzy dissimilarity is computed by $\eta_{L_F/L_M}=1-\bar{\eta}_{L_F/L_M}$.

The decision criterions of fuzzy dissimilarity and fuzzy similarity for all fluxes and metabolite-flows in GSMN are summed up all two-side membership functions, and respectively expressed as follows:

$$\eta_{L_F}=\sum_{i=1}^{N_F}\eta_{L_{F_i}}\left(L_{F_i}\right)\Big/N_F \quad \text{(S1-22)}$$

$$\eta_{L_M}=\sum_{j=1}^{N_M}\eta_{L_{M_j}}\left(L_{M_j}\right)\Big/N_M \quad \text{(S1-23)}$$

$$\eta_{v}=\sum_{k=1}^{N_F}\eta_{k}\left(v_{k}\right)\Big/N_F \quad \text{(S1-24)}$$

$$\eta_M = \sum_{m=1}^{N_M} \eta_m(r_m) \Big/ N_M \qquad \text{(S1-25)}$$

- Each objective in the IACT problem is attributed to a membership function. Each membership grade is between zero and one. The value of zero implies that the objective is completely unsatisfied. In contrast, the objective fulfils so that the membership grade achieves one. Using the intersection of the membership functions, the multi-objectives can formulate as maximization of a hierarchical fitness, η_D, as

$$\eta_D = \left(\eta_{CV} + \min\{\eta_{CV}, \eta_{DV}\}\right)/2 \qquad \text{(S1-26)}$$

where the first priority grade (η_{CV}) in the hierarchical fitness considered the membership grades for the cell growth rates of the cancer and perturbed cells, and computed by the mean-min evaluation for both cells as follows

$$\eta_{CV} = \left(\left(\eta_{CV}^{CA} + \eta_{CV}^{PB}\right)/2 + \min\{\eta_{CV}^{CA}, \eta_{CV}^{PB}\}\right)/2 \qquad \text{(S1-27)}$$

It accounts for the membership grade, η_{CV}^{CA}, for the cell growth of cancer cells (the first goal) and cell viability grade, η_{CV}^{PB}, of perturbed cells (second goal in Eq.(S1-6)).

The second priority grade, η_{DV}, in the hierarchical fitness is to evaluate metabolic deviation of perturbed cells (third goal and fourth goal) by the mean-min evaluation as

$$\eta_{DV} = \left(\left(\eta_{S_F} + \eta_{S_M} + \eta_{L_F} + \eta_{L_M} + \eta_v + \eta_M\right)/6 + \min\{\eta_{S_F}, \eta_{S_M}, \eta_{L_F}, \eta_{L_M}, \eta_v, \eta_M\}\right)/2$$

(S1-28)

The hierarchical fitness implies that the first priority grade is more priority than the second one (A numerical example in Table S1-2), and is applied as a fitness evaluation in the NHDE algorithm for determining the next better individuals.

Table S1-2. A numerical example to illustrate the hierarchical decision fitness

Case	η_1	η_2	$\min\{\eta_1, \eta_2\}$	η_D
**1**	1	0	0	0.5
**2**	0.6	0.4	0.4	0.5
**3**	0.4	0.6	0.4	0.4

Figure S2: The restrictions on the bounds of the inner optimization problem.

The IACT framework is not only to discover modulated reactions based on gene-centric approach, but also suits for metabolite-centric and reaction-centric approaches. The approach depends on the constraint on the lower and upper bounds of modulated reactions in the inner optimization problems. For gene-centric approach, the lower and upper bounds of modulated reactions for gene-centric approach are constrained as following:

$$
\begin{aligned}
&\text{Up-regulation:}\\
&\begin{cases}
(1-\delta)v_{f,i}^{basal} + \delta v_{f,i}^{UB} \le v_{f,i} \le v_{f,i}^{UB} \\
v_{b,i}^{LB} \le v_{b,i} \le (1-\delta)v_{b,i}^{basal} + \delta v_{b,i}^{LB};\ z_i \in \Omega^{TR}
\end{cases}\\
&\text{Down-regulation:}\\
&\begin{cases}
v_{f,i}^{LB} \le v_{f,i} \le (1-\delta)v_{f,i}^{basal} + \delta v_{f,i}^{LB} \\
(1-\delta)v_{b,i}^{basal} + \delta v_{b,i}^{UB} \le v_{b,i} \le v_{b,i}^{UB};\ z_i \in \Omega^{TR} \backslash \Omega^{IZ}
\end{cases}\\
&\begin{cases}
v_{f,i}^{LB} \le v_{f,i} \le v_{f,i}^{UB} \\
v_{b,i}^{LB} \le v_{b,i} \le v_{b,i}^{UB};\ z_i \in \Omega^{TR} \cap \Omega^{IZ}
\end{cases}\\
&\text{Knockout:}\\
&\begin{cases}
v_{f,i} = 0 \\
v_{b,i} = 0;\ z_i \in \Omega^{TR} \backslash \Omega^{IZ}
\end{cases}\\
&\begin{cases}
v_{f,i}^{LB} \le v_{f,i} \le v_{f,i}^{UB} \\
v_{b,i}^{LB} \le v_{b,i} \le v_{b,i}^{UB};\ z_i \in \Omega^{TR} \cap \Omega^{IZ}
\end{cases}
\end{aligned} \tag{S2-1}
$$

where Ω^{IZ} is the set of reactions regulated by isozymes determined using the GPR associations, and modulation parameter, δ, is determined by the NHDE algorithm. The metabolite-centric regulators are considered towards modulating the synthesis reactions flowing to the active metabolites. The lower and upper bounds of modulated reactions for the ith active metabolite are restricted as

Regulated bounds for the ith active metabolite:

Up-regulation:

$$\begin{cases} (1-\delta)v_{f,j}^{CA} + \delta v_{f,j}^{UB} \le v_{f,j} \le v_{f,j}^{UB}; j \in N_{ij} > 0 \text{ and } j \in \Omega^{rxn} \\ (1-\delta)v_{b,j}^{CA} + \delta v_{b,j}^{UB} \le v_{b,j} \le v_{b,j}^{UB}; j \in N_{ij} < 0 \text{ and } j \in \Omega^{rev} \end{cases}$$

Down-regulation: (S2-2)

$$\begin{cases} v_{f,j}^{LB} \le v_{f,j} \le (1-\delta)v_{f,j}^{CA} + \delta v_{f,j}^{LB}; j \in N_{ij} > 0 \text{ and } j \in \Omega^{rxn} \\ v_{b,j}^{LB} \le v_{b,j} \le (1-\delta)v_{b,j}^{CA} + \delta v_{b,j}^{LB}; j \in N_{ij} < 0 \text{ and } j \in \Omega^{rev} \end{cases}$$

Knockout:

$$\begin{cases} v_{f,j} = 0; j \in N_{ij} > 0 \text{ and } j \in \Omega^{rxn} \\ v_{b,j} = 0; j \in N_{ij} < 0 \text{ and } j \in \Omega^{rev} \end{cases}$$

where N_{ij} is the stoichiometric coefficient for the ith metabolite and j^{th} reaction, Ω^{rxn} is the set of overall reactions in the metabolic model, Ω^{rev} is the set of reversible reactions.

Figure S3: Introduction to the Nested Hybrid Differential Evolution (NHDE) algorithm.

The multi-objectives in the outer optimization can be converted as a maximization problem of a hierarchical decision-making fitness, η_D. The inner optimization problems consist of FBA problems for treatment and perturbation are linear programming (LP) and UFD problems are quadratic programming (QP). The IACT platform can be rewritten as the following generalized formulation for easily explaining the NHDE algorithm.

$$\begin{cases} \text{Outer optimization problem:} \\ \quad \max f(\mathbf{x},\mathbf{z}) = \eta_D \\ \text{subject to the inner optimization problems:} \\ \text{(1) LP and QP problems for treating cancer cells.} \\ \text{(2) LP and QP problems for perturbing normal cells} \end{cases} \quad \text{(S3-1)}$$

The NHDE algorithm is a stochastic optimization based on hybrid differential evolution (HDE), which was extended from the original DE algorithm (Storn and Price, 1996; Storn and Price, 1997). The basic operations of original DE and NHDE are shown in Tab. S1-1. The detailed procedures have discussed by Wang (2017).

Table S3-1. Basic operations for the original DE and NHDE algorithms

**Original DE**	**NHDE**
1. Representation and initialization<br>2. Mutation<br>3. Crossover operation<br>4. Selection and evaluation<br>5. Repeat steps 2 to 4	1. Representation and initialization<br>2. Mutation with rounding operation<br>3. Crossover operation<br>4. Restriction operation<br>5. Selection and evaluation<br>6. Solve LP/QP problems for each candidate<br>7. Compute fitness for each feasible design<br>8. Migration operation performed naturally or enforced if necessary<br>9. Repeat steps 2 to 6

The NHDE algorithm is a parallel direct search procedure as shown in Table S1-2 that is a modified version from Wang (2017; 2020) to suit for solving the IACT problem. NHDE utilizes a population of N_p individuals (enzymes, metabolites and reactions) to

find an optimal solution. The initialization process randomly generates N_p individuals $\mathbf{z}_i$ with corresponding modulation actions to cover the entire search space uniformly. Each population consists of a set of enzymes, metabolites, or reactions that depend on modulation using gene-centric, metabolite-centric or reaction-centric approach.

Table S3-2. The NHDE algorithm for iteratively selecting a set of candidates towards discovering anticancer targets

NHDE

1. Representation and initialization

   $$(\mathbf{z}^0)_i = \text{uniformInt }(\mathbf{z}^{\min}, \mathbf{z}^{\max}), i = 1,..., N_p$$

   Each individual is generated by an integer random number between $\mathbf{z}_{\min}$ and $\mathbf{z}_{\max}$ with uniform distribution
2. Mutation with rounding operation

   $$(\hat{\mathbf{z}}^G)_i = \text{INT}\left\{(\mathbf{z}^G)_p + \rho^G\left[(\mathbf{z}^G)_j - (\mathbf{z}^G)_k + (\mathbf{z}^G)_l - (\mathbf{z}^G)_m\right]\right\}$$
3. Crossover operation

   $$z_{ji}^G = \begin{cases} z_{ji}^{G-1}, & \text{if a random number} > C_R \\ \hat{z}_{ji}^G, & \text{otherwise}, j = 1,...,n; i = 1,..., N_p \end{cases}$$
4. Restriction operation

   $$z_{ji}^G = \begin{cases} z_{ji}^G, z_{ji}^G \in [z_j^{\min}, z_j^{\max}] \\ \text{uniformInt}(z_j^{\min}, z_j^{\max}), z_{ji}^G \notin [z_j^{\min}, z_j^{\max}] \end{cases}$$
5. Selection and evaluation
   (a) For each candidate, solve the LP/QP problems for treatment of cancer cells, respectively.
   (b) Compute penalty if the above problems are infeasible; otherwise, executes the next step.
   (c) For each optimal solution from Step (a), solve LP/QP problems for perturbation of normal cells, respectively.
   (d) Compute penalty if the above problems are infeasible, and then performs the next step.
   (e) Compute fitness for each feasible solution
   $$fitness = f(\mathbf{x}, \mathbf{z}) + \text{penalty}$$
6. Migration operation performed naturally or enforced if necessary

   $$(\mathbf{z}^G)_i = \text{uniformInt }(\mathbf{z}^{\min}, \mathbf{z}^{\max}), \text{if } \zeta \le \varepsilon = [0,1]$$

7.	Repeat steps 2 to 6

References

Storn, R. and Price, K. (1996) Minimizing the real functions of the ICEC'96 contest by differential evolution. Evolutionary Computation, 1996., Proceedings of IEEE International Conference on. IEEE, Nagoya, pp. 842 - 844.

Storn, R. and Price, K. (1997) Differential evolution - A simple and efficient heuristic for global optimization over continuous spaces, Journal of Global Optimization, 11, 341-359.

Wang, FS (2017) Nested differential evolution for mixed-integer bi-level optimization for genome-scale metabolic networks, Ch.12, in Differential evolution in chemical engineering edited by Rangaiah and Sharma, World Scientic.

Wang, F.S.; Wu, W.H.; Hsiu, W.S.; Liu, Y.J.; Chuang, K.W. (2020). Genome-scale metabolic modeling with protein expressions of normal and cancerous colorectal tissues for oncogene inference. Metabolites, 10, 16

Hsu, KC and Wang, FS. (2013). Fuzzy optimization for detecting enzyme targets of human uric acid metabolism. Bioinformatics. 29(24) 3191-3198.

Zimmermann, HJ. (2010). Fuzzy set theory. Advanced Review. 2, 317-332.

Massad, E; Siqueira Ortega, NR; Carvalho de Barros, L; José Struchiner, C. (2008). Fuzzy logic in action: Applications in epidemiology and beyond. Springer, Springer-Verlag Berlin Heidelberg
